# Toward interpretable expected goals modeling using Bayesian mixed models

**DOI:** 10.3389/fspor.2025.1504362

**Published:** 2025-04-23

**Authors:** Loïc Iapteff, Sebastian Le Coz, Maxime Rioland, Titouan Houde, Christopher Carling, Frank Imbach

**Affiliations:** ^1^ Seenovate, Montpellier, France; ^2^ Université de Lyon, Lyon2, Bron, France; ^3^ Laboratoire Sport, Expertise and Performance INSEP, Paris, France; ^4^ DMeM, Univ Montpellier, INRAe, Montpellier, France

**Keywords:** soccer, expected goals, Bayesian inference, generalized linear mixed model, transfer learning

## Abstract

Empowered by technological progress, sports teams and bookmakers strive to understand relationships between player and team activity and match outcomes. For this purpose, the probability of an event to succeed (e.g., the probability of a goal to be scored, namely, xG for eXpected Goals) provides insightful information on team and player performance and helps statistical and machine learning approaches predict match outcomes. However, recent approaches require powerful but complex models that need more inherent interpretability for practitioners. This study uses a Bayesian generalized linear mixed-effects model to introduce a simple and interpretable xG modeling approach. The model provided similar performance when compared to the StatsBomb model (property of the StatsBomb company) using only seven variables relating to shot type and position, and surrounding opponents (AUC = 0.781 and 0.801, respectively). Pre-trained models through transfer learning are suitable for identifying teams’ strengths and weaknesses using small sample sizes and enable interpretation of the model’s predictions.

## 1 Introduction

Football is a globally popular sport and its financial and social impact attracts researchers whose main aim is to increase comprehension of training and match-play performance (1–3). Thanks to technological and analytical evolutions, new performance-oriented research perspectives have emerged from the analysis of player performance. Both training and match data, collected from football players using global navigation satellite systems (4) and markerless optical tracking systems (5), have become more plentiful and increasingly accurate. The information enables the development of advanced statistical and machine learning approaches to help analyze and subsequently optimize football performance and attempt predictions of match outcomes.

A popular performance metric in football is expected goals (xG). This metric represents the probability of a shot resulting in a goal. It was first introduced in football by Green (6) with the aim of identifying the key factors underpinning how goals are scored and has become a valuable objective measure of an individual player’s performance that can also be extended to the team level (7–10). To date, xG models typically account for spatio-temporal information, such as the time, the distance and angle between the player and the goal at the shooting time, the type of shot, and the preceding event (i.e., the last action such as a low pass or a high pass). Beyond these data, previous studies have reported different approaches and model architectures (11–16). Modeling xG requires multiple features as the complexity of the task and its variability calls into question its predictability when using a restricted set of game features (e.g., the goal distance) (15). However, Umami et al. (16) reported that a logistic regression using only a few features (the distance and angle to the goal, and whether the shot is headed or not) provided convincing results. Alternatively, more complex architectures have been employed to attempt to better estimate xG (14). In effect, the authors in the latter study compared a logistic regression with non-linear ensemble learning algorithms (random forest and adaptive boosting) to predict the match score by summing the estimated xG of each shot opportunity. According to their results, the random forest algorithm provided the best model performance. In another study, Anzer and Bauer (11) used advanced features such as the height of the ball when the shot was attempted and analysis of the player’s movement at the time of the shot. By comparing several supervised machine learning models, a gradient boosting model that accounted for the type of shot (header, leg kick, and direct free kick) provided the best performance in predicting the number of goals scored.

Most studies that have attempted to model xG have focused on fitting the best model using non-linear and complex model architectures, at the expense of model interpretability. These studies aimed to achieve the best performance in predicting that the shot will be converted into a goal. However, complex models are difficult to interpret. There are methods for explaining such models, such as the use of Shapley values (17, 18), but these approaches are still criticized today (19, 20), questioning the ability of one to fully master the complexity of Shapley value calculations. There are a few studies that have focused on xG modeling while preserving interpretability. To build a model that identifies key factors influencing xG, Decroos and Davis (13) and Bransen and Davis (12) proposed the use of a generalized additive model (GAM). The studies show that GAMs provide comparable results to a more complex gradient boosting model while retaining the advantage of interpretability. To further improve the interpretability of the model and since logistic regression has proved effective in modeling xG (16), one should consider the relationships between features and xG to be linear and consequently should utilize a generalized linear model instead of a GAM. One also assumes that soccer games evolve and that patterns are slightly different across seasons and competitions. As such, the present authors made a choice to investigate a Bayesian framework with mixed effects. Very recently, Scholtes and Karakuş (21) also proposed a hierarchical Bayesian approach to model xG. They used this model to determine whether the individual player or their positional role impacted xG. The main strength of their work was the identification of specific player abilities throughout an interpretable model and a rigorous assessment of prediction uncertainty. However, the potential of the Bayesian framework was arguably not fully exploited and further research is warranted.

In the present article, an alternative prior specification method is proposed to model xG: an interpretable Bayesian generalized linear mixed-effects model (22). This model might achieve better estimation quality, and its benefits are potentially numerous. First, the linear structure makes the model very easy to interpret by analyzing the model’s coefficients. Second, the inclusion of random effects means that intra- and inter-player/team variability can be considered. This enables interpretation of the strength of players and/or teams in specific game situations. Third, using the Bayesian framework enables the utilization of limited training data while incorporating expert prior knowledge. Such a model is not new and is used in other fields and applications. Yet, it was unseen in xG modeling until the recent work of Scholtes and Karakuş (21) and also demonstrates the aforementioned advantages. In our study, we propose to further exploit the Bayesian contribution with a transfer learning strategy to build a highly informative prior. The prior is built using past competition, rather than relying on a hand-constructed prior, which is inevitably less informative. A study on the impact of prior choice on the posterior was presented using the Wasserstein Impact Measure (WIM) (23), showing the benefits of choosing the prior we propose (more informative prior and better predictive performance). Furthermore, a practical model explainability of outcomes is provided through Shapley Additive exPlanations estimates (SHAP), which can, according to the literature (24), benefit football analysts and coaches in their decision-making processes.

Following the presentation of the data and the features used to evaluate xG (Section 2.1), we will define our xG model (Section 2.2) and then present the results (Section 3). Thereafter, we will engage in a discussion of the results and benefits of the model within the context of the existing literature (Section 4).

## 2 Material and methods

### 2.1 Dataset description

**Figure 1:**
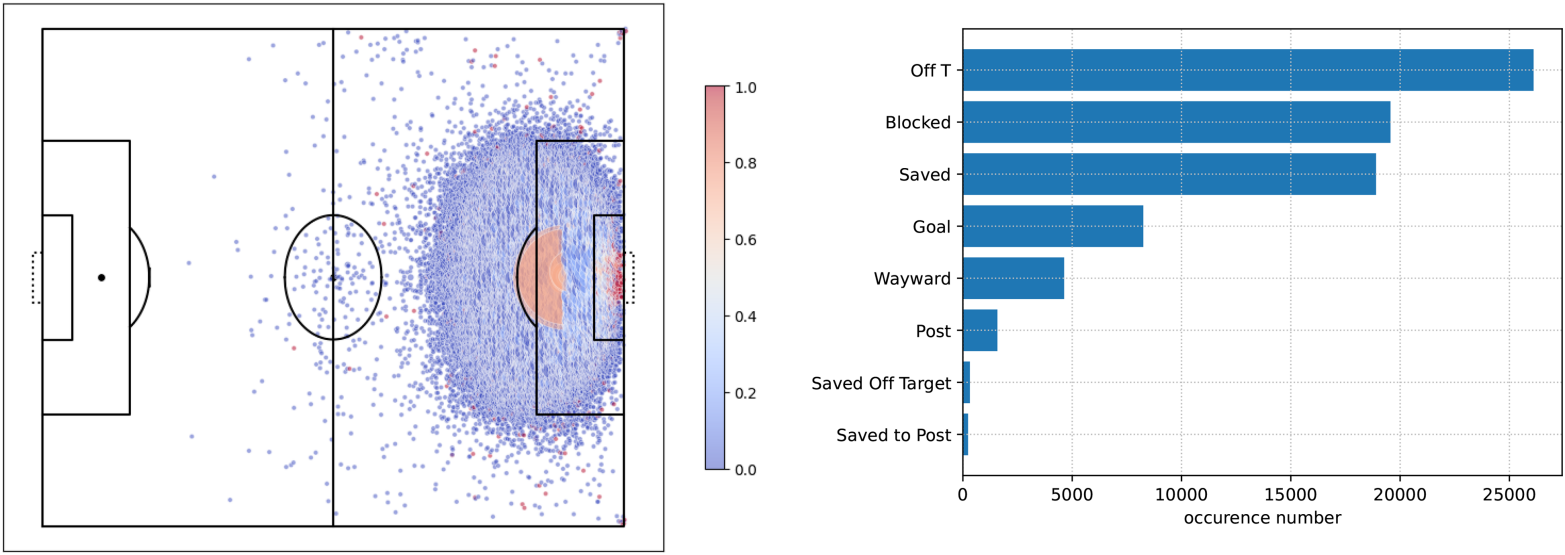
Shot outcomes. On the left, dots represent shot locations on the field and the gradient color denotes the frequency of scored shots per location. On the right, all outcomes and their occurrences are listed.

Here, we used the StatsBomb open dataset (25) that includes 460 matches with 63,177 shots from 11 different competitions including the FIFA World Cup, Women’s World Cup, UEFA Euro, UEFA Women’s Euro, Indian Super League, NWSL, and Premier League between 2003 and 2022. Among these shots, we retained 59,417 from open-play situations and discarded 3,760 from set-play actions (penalty, free kick, or corner). Hence, only shots from open play were considered. Shot location and occurrence is displayed in Figure 1.

Shot data include the ball’s location, the player’s location, and the time of the shot. From these spatio-temporal data, we extracted features for subsequent modeling. An exhaustive list of the features is provided in Table 1. Many of these features are well-known and are common to previous studies (11, 26). However, subtle features such as the *Best_angle* may provide relevant information. Since our dataset includes quantitative and qualitative features (*Position, Body_part*, and *Last_action*), the latter have been one-hot encoded for the modeling. To reduce dimensionality and prevent collinearity, we performed a feature selection as described in Section 2.2.

**Table 1:** Feature descriptions and value range.

Feature	Description	Values
Minute	Time of the shot in minutes	[0,128]
Distance	The distance between the location of the shot and the center of the goal	[0.4,93]
Angle	The angle of the shot (ball, center of the cage, and center of the field). Its value is 0 when the shot is aligned with the center of the cage and 90 when the shot is from the back line.	[0,90]
Distance_goalkeeper	The distance between the goalkeeper and the center of the goal	[0,118]
Goalkeeper_on_traj	If the goalkeeper is on the trajectory (in the triangle formed by the two posts and the shot location)	{1, 0} for {True, False}
Nb_opponent_on_traj	The number of opponents, excluding the goalkeeper, on the trajectory	[0,10]
Closest_opponent	The distance between the shot location and the closest opponent	[0,92]
Opponent_nearby	The number of opponent closer than 3 m	[0,9]
Teammate_front	The number of teammates more advanced in the field	[0,10]
Best_angle	The best shot angle (left post, shot location, or right post angle if there is neither opponent nor goalkeeper on the trajectory, else the largest space)	[0,180]
Under_pressure	Statsbomb feature estimating whether the shooter is under pressure or not during the shot	{1, 0} for {True, False}
Position	The position of the shooter	e.g., “Goalkeeper,” “Right Attacking Midfield,” “Center Forward,” … 25 different instances
Body_part	The part of the body used for the shot	{“Left Foot,” “Right Foot,” “Head,” or “Other”}
Last_action	The action preceding the shot	{“Regular Play,” “From Throw In,” “From Keeper,” “From Corner,” “From Counter,” “From Free Kick,” “From Goal Kick,” or “From Kick Off”}

The StatsBomb dataset provides an estimate of xG, which we will refer to as StatsBomb xG. Their model, which remains undetailed, has most likely been trained on their full dataset of matches instead of the open-source dataset used in our study. Furthermore, some features such as shot impact height are used and not shared in the open-source data set. This StatsBomb xG estimate, which has proven its performance (27, 28), will be considered as a baseline model for comparison.

### 2.2 Model definition

In the literature, many statistical and machine learning methods have been tested to model xG (presented in Section 1), most of them focusing on the best predictive performance at the expense of model interpretability. In this study, we propose a model allowing a detailed analysis of the shot quality. We built a Bayesian logistic regression with mixed effects, defined in Equations 1 and 2. Normal and Gamma prior distributions (see Equation 3) have been set for fixed and random effects, with adjusted means and standard deviations accordingly (the method is described hereafter).

Let us define $x_{i}$ and $y_{i}$, respectively, as the vector of feature values and the shot result for observation i ($y_{i}\in \{0,1\}$, 1 represents a goal and 0 otherwise). Hence, we define $y_{i_{k}}$ such that the i predicted xG for a team k in the following:

(1) $$y_{i_{k}}=(1+e^{-\theta_{i_{k}}}{)}^{-1}+\epsilon_{i_{k}},\;\epsilon_{i_{k}}∼N(0,\sigma_{i}^{2}),$$

(2) $$\theta_{i_{k}}=\alpha +\sum_{\;j=1}^{p}(\beta_{j}+\eta_{i}^{\left(\beta_{j}\right)})x_{i_{k}j}+\eta_{i}^{\left(\alpha \right)},\;\eta_{i}^{\left(q\right)}∼N(0,\omega_{q}^{2}),$$

(3) $$\begin{array}{rl} \alpha & ∼N(\mu_{\alpha },\sigma_{\alpha }^{2}),\;\beta ∼N(\mu_{\beta },\Sigma_{\beta }),\;\omega_{q}∼\Gamma (a_{q},b_{q}),\\ \sigma & ∼\Gamma (a,b) \end{array}.$$

where $q\in \{\alpha ,\beta_{1},\ldots ,\beta_{p}\}$ are the fixed effects parameters to be identified, p is the number of features, $i\in \{1,\;\ldots ,\;N\},\;k\in \{1,\ldots \;,\;n_{i}\}$, N is the number of teams, and $n_{i}$ is the number of observations for team i. Moreover, the parameters $q\in \{\alpha ,\beta_{1},\ldots ,\beta_{p}\}$ on which random effects $\eta_{i}^{\left(q\right)}$ are applied must be determined. The model selection is performed using Pareto-smoothed importance sampling-leave-one-out (PSIS-LOO) (29). This Bayesian approach to model selection enables us to select the model that offers the best predictive accuracy in a robust way thanks to its leave-one-out strategy. In addition, the combination of Hamiltonian Monte Carlo and approximate cross-validation enables highly efficient model fitting and score estimation for a wide range of model selection contexts (30). Thus, not all $\eta_{i}^{\left(q\right)}$ will be considered a random variable but some will be fixed to 0.

The choice of the prior distribution is a crucial step in the model fitting process (choice of $\mu_{\alpha },\sigma_{\alpha },\mu_{\beta },\Sigma_{\beta },a_{q},b_{q},a,b$). Alternatively, two different approaches can be considered instead of a non-informative prior, with (i) a prior identification from expert knowledge, and (ii) a prior identification using a *baseline* model, computed on different data. Scholtes and Karakuş (21) used the first method while the latter method has been employed in this study, thus considering a different training dataset. Hence, estimating prior distributions using a separate dataset ensures unbiased model training and model generalization. Our method therefore uses a transfer learning strategy (i.e., the reuse of a set of functions or knowledge learned from a source task). A model is fitted for each competition, the prior being built from the other competitions. In other words, let us assume that c is the competition of interest and that $\bar{c}$ are the other competitions available in our dataset. The $\bar{c}$ are used to fit a model M without informative prior knowledge. The model of interest, fitted on c, then benefits from the posterior knowledge identified in M.

A Bayesian model with flat prior and without random effect was fitted on the shots from $\bar{c}$. The posterior distribution obtained is then used to build the prior for the model on competition c, and is defined as

$$\alpha ∼N({\hat{\alpha }}_{\bar{c}},g.{\hat{\sigma }}_{\alpha_{\bar{c}}}^{2}),\;\beta ∼N({\hat{\beta }}_{\bar{c}},g.{\hat{\Sigma }}_{\beta_{\bar{c}}}).$$

The coefficient g is chosen so that the mean of the standard deviations for the fixed effects is equal to 0.5 to ensure that the prior information is neither too strong nor too weak. Regarding the parameters σ and $\omega_{q},q\in \{\alpha ,\beta_{1},\ldots ,\beta_{p}\}$, the priors were fixed to $\sigma ∼\Gamma (1,0.5),\omega_{q}∼\Gamma (0.3,0.1)$. The variance of σ prior distribution is large, while $\omega_{q},q\in \{\alpha ,\beta_{1},\ldots ,\beta_{p}\}$ is more restrictive. This allows us to avoid the high values obtained when teams are under-represented in the studied competition, and when observations are not necessarily representative of actual team performance.

Posterior distributions have been estimated through Markov chain Monte Carlo with a Hamiltonian Monte Carlo algorithm [No-U-Turn Sampler, (31)]. The No-U-Turn sampler allows us to simplify the tuning of the standard Hamiltonian Monte Carlo method with a similar efficient performance. Then, on the basis of the sampling obtained, we used 10,000 iterations and four chains, with a burn-in period of 1,000 samples for sampling. All the analyses were conducted using the latest version of the Python library PyMC (32). Once the sampling from the posterior distribution was complete, the model’s parameters were estimated according to the maximum *a posteriori* for further predictions.

For a given competition, 70% of the shots were used as a training set while the remaining 30% were used as a test set. All the results presented in Section 3 stem from the test set. Model selection was carried out using PSIS-LOO for each competition to select q parameters on which random effects are applied. Consequently, the random effect structure could vary across competitions.

As the work of Scholtes and Karakuş (21) is analogous to ours, we also fitted their model to our data and features (selected in Section 2.3) to emphasize the benefits of using an informative prior in small datasets. The following priors presented in this section correspond to the priors used to reproduce their work. The same features used to fit our model were considered, but the priors were chosen as proposed in their paper. Since some features are shared between our respective works, the priors chosen for the associated parameters are the identical (N stands for normal distribution and SN for skew-normal distribution):

• intercept : N(μ=0,σ=5)
• distance_goalkeeper : N(μ=0,σ=5)
• distance : SN(μ=−1,σ=5,α=−1)
• body_part_head : N(μ=0,σ=5)
• nb_opponent_traj : SN(μ=−1,σ=5,α=−1)

For the other features, we applied the same method for their selection and finally used the following priors:

• best_angle : SN(μ=1,σ=5,α=1)
  This feature is similar to the shot_angle feature used in Scholtes and Karakus’ paper, which is the left post, shot location, and right post angle. The prior is therefore the same as that used in their article for the shot_angle feature.
• angle : SN(μ=−1,σ=5,α=−1)
  The angle we use represents alignment with the goal. A higher value represents a less centered positioning, which translates into a lower score probability and explains the choice of μ and α.
• closest_opponent : SN(μ=1,σ=5,α=1)
  μ=1 and α=1 because the closer the opponent, the greater the pressure.

In the following, we will refer to this model as Scholtes’ model.

### 2.3 Feature selection and performance criteria

**Figure 2:**
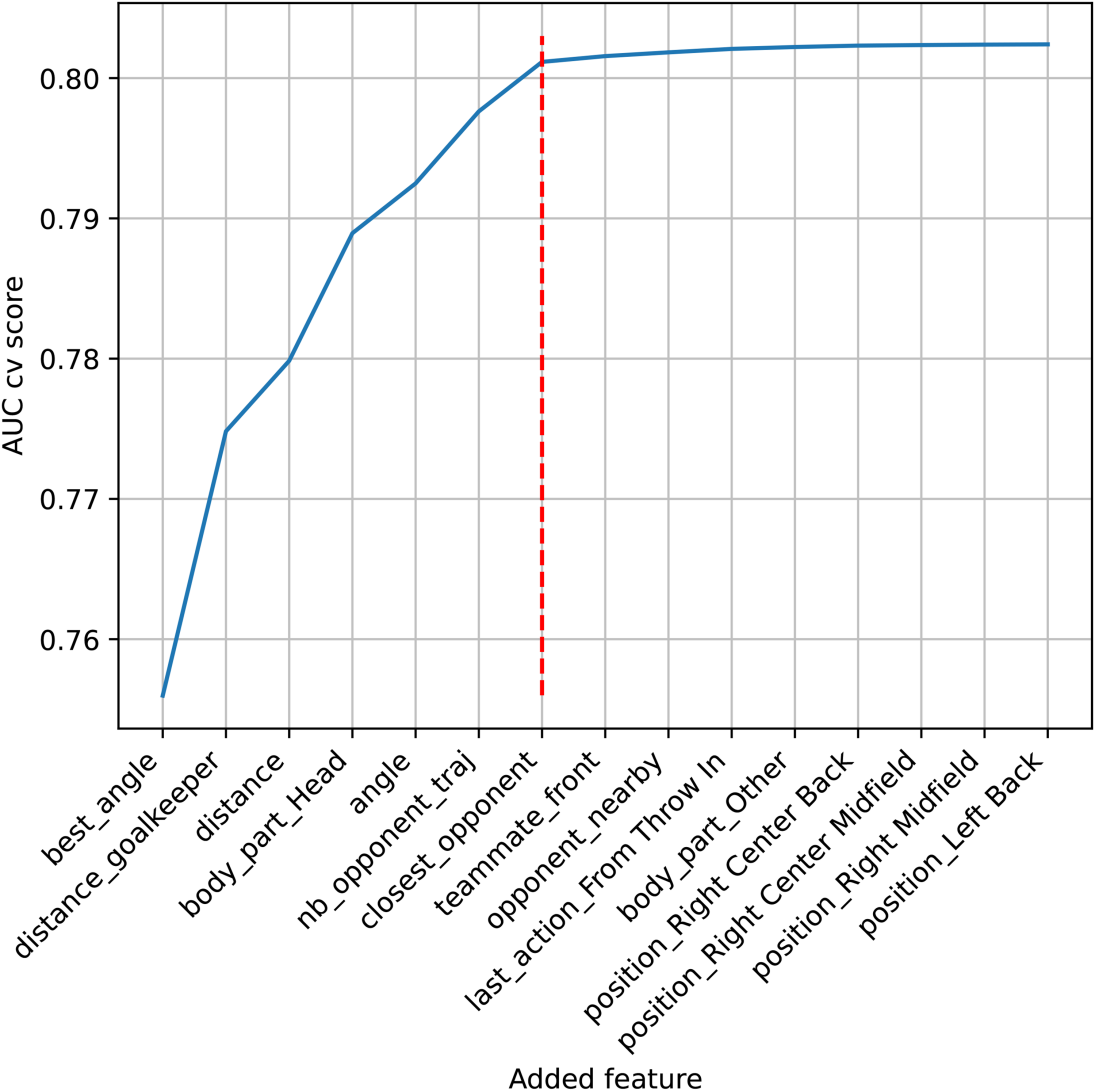
AUC score evolution of the fivefold cross-validation. On the *x*-axis, the best features are added one by one. The red dotted line represents the last selected feature, where the AUC reaches a plateau.

To use our approach, the model used to build the prior must be fitted using the same features as the model of interest. Thus, feature selection was conducted using the training set of all competitions in a *global* model. A frequentist logistic regression was performed, discarding random effects and any prior knowledge. For feature selection, we performed a forward selection starting with the best features to model xG and then added features one by one until the area under the curve (AUC) reached a plateau (see Figure 2). At each step, all unused features were tested.

Seven features were selected from the fivefold cross-validation (Figure 2), with (1) best_angle, (2) distance_goalkeeper, (3) distance, (4) body_part_head (one-hot-encoded feature, 1 if the shooter struck the ball with their head, 0 otherwise), (5) angle, (6) nb_opponent_traj, and (7) closest_opponent. They constitute the set of predictors for subsequent modeling.

The area under the curve, balanced accuracy, precision, recall, specificity, and F1 score were used to measure the efficiency of the models.

Each competition was used for model fitting, leading to models with heterogeneous fixed parameter estimates based on the prior information.

## 3 Results

The comparison of the performance of the proposed model (i.e., a Bayesian mixed effect logistic regression, namely, *Bayesian xG*) with that of StatsBomb’s and Scholtes’ models is reported in Table 2. Considering all competitions, the results show that model performances differed slightly, but similar performance was obtained with the Bayesian approach and the StatsBomb model. Scholtes’ model seemed to suffer from less informative priors when fitted on small data samples. This is supported by a lower AUC score than the other models, and the xG predictions tend to be symptomatically higher (higher recall with a smaller threshold).

**Table 2:** Model performance of the Bayesian model, StatsBomb xG, and Scholtes’ model.

Threshold	Score	Bayesian xG	StatsBomb xG	Scholtes xG
	AUC	0.781	0.801	0.578
	Balanced accuracy	0.517	0.520	0.553
	Precision	0.754	0.845	0.159
0.8	Recall	0.036	0.041	0.238
	Specificity	0.999	0.999	0.868
	F1 score	0.068	0.078	0.191
	Balanced accuracy	0.559	0.558	0.504
	Precision	0.563	0.608	0.095
0.5	Recall	0.129	0.124	0.944
	Specificity	0.988	0.991	0.064
	F1 score	0.211	0.207	0.173
	Balanced accuracy	0.710	0.735	0.502
	Precision	0.228	0.250	0.095
0.2	Recall	0.687	0.716	0.996
	Specificity	0.733	0.754	0.007
	F1 score	0.344	0.372	0.173

The results are displayed according to three thresholds.

Having obtained a reasonable model, we will now illustrate the advantages of the proposed approach and focus on a given international football competition: the FIFA World Cup 2022. First, to underline the impact of the prior used for the *Bayesian xG* model compared to the prior proposed by Scholtes, we calculated the WIM. This measure is used to compare two distributions, and in the Bayesian framework, to compare two posterior distributions obtained with distinct priors. As suggested in the original paper, we computed the WIM using the uniform prior. The aim of the approach is to evaluate the quantity of information provided by the prior: the higher the WIM between a given prior and the uniform prior, the more informative the prior is. The WIM was computed using the samples obtained with the MCMC algorithm, and resulted in a WIM almost four times higher for the *Bayesian xG* prior against the uniform prior than for the Scholtes prior against the uniform prior (Table 3).

**Table 3:** Wasserstein Impact Measure using the priors proposed for the Bayesian model and Scholtes’ model and using a uniform prior for all parameters.

	Uniform prior	Scholtes’ prior
Bayesian xG prior	0.69	0.66
Scholtes’ prior	0.18	

**Figure 3:**
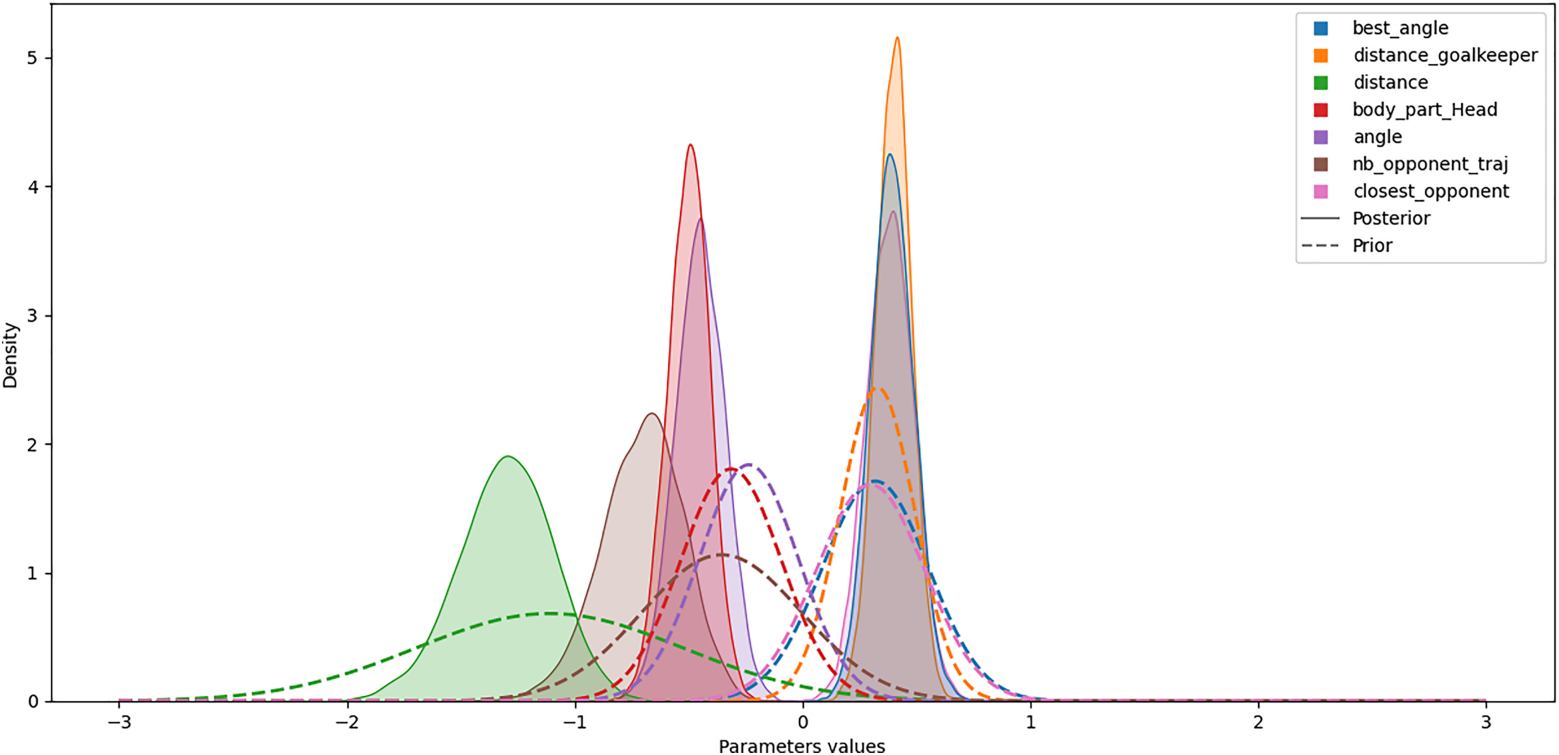
Univariate posterior and prior distribution of the β parameters for the 2022 FIFA World Cup.

To focus on the *Bayesian xG* model, the maximum *a posteriori* parameter estimation showed that the *distance* feature (i.e., the distance between the shot location and the center of the goal) had the greatest negative influence on the predicted xG (see Table 4). Furthermore, the optimal model structure retained for xG prediction includes a random intercept $\omega_{\alpha }$ and two random slopes (on *angle* and *closest_opponent* parameters, denoted by $\omega_{\beta_{5}}$ and $\omega_{\beta_{7}}$, respectively). Since $\omega_{\beta_{5}}$ and $\omega_{\beta_{7}}$ differ from 0, they highlight team-specific traits and relationships between these predictors and the predicted xG. Accordingly, such a model structure allows for consideration of inter-team variability. The univariate posterior and prior distribution for the fixed effects showed the differences between the Bayesian model calibrated on the 2022 FIFA World Cup and the *global* model fitted on other competitions (see Figure 3). For this competition, the features *nb_opponent_on_traj, angle*, and *body_part_head* had a particularly higher negative impact than the *global* model. This means that in this competition and situation, the players were less likely to score than usual. However, the optimal model retained a random slope on the *angle* feature, meaning that some teams were significantly better than others to deal with the bad angles.

**Table 4:** Maximum *a posteriori* parameter estimation for the FIFA World Cup 2022.

α	$\beta_{1}$	$\beta_{2}$	$\beta_{3}$	$\beta_{4}$	$\beta_{5}$	$\beta_{6}$	$\beta_{7}$	$\omega_{\alpha }$	$\omega_{\beta_{5}}$	$\omega_{\beta_{7}}$
−2.58	0.393	0.397	−1.296	−0.501	−0.449	−0.703	0.378	0.427	0.364	0.326

The beta’s are defined as the parameters for (1) best_angle, (2) distance_goalkeeper, (3) distance, (4) body_part_head, (5) angle, (6) nb_opponent_traj, and (7) closest_opponent.

**Figure 4:**
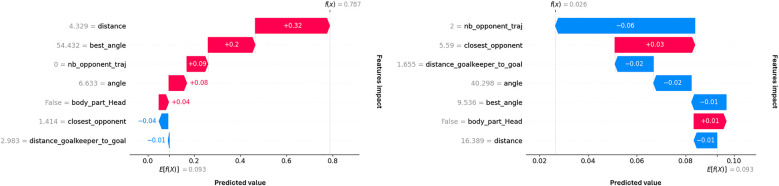
SHAP waterfall plots for two separate shots. The shot on the left has a high Bayesian xG prediction (0.787) and was missed, the one on the right has a low Bayesian xG prediction (0.026) and was scored.

Computing SHAP values allowed us to explain the model predictions and, for a given shot, to interpret the impact of each feature’s value on the prediction. In addition, we can examine poorly predicted shots to understand where the failure arises from. For example, Figure 4 presents two poorly predicted shots and their respective feature contributions. The first shot has a very high xG value because it is close, central, and there is no opponent on its trajectory. However, the pressure exerted by the nearest opponent likely exceeded the model’s prediction, resulting in a missed shot. The second shot was challenging, characterized by its considerable distance and lateral displacement, with two opposing players present on its trajectory. Nevertheless, the player successfully converted his attempt into a goal.

## 4 Discussion

The present xG mixed-effect model, based on a dataset from the 2022 FIFA World Cup, provided an opportunity to study teams’ goal scoring through strengths and weaknesses in an interpretable and explainable way. The results are arguably of major interest to coaching practitioners, sports scientists, and researchers interested in determining the influence of player and team actions on scoring goals and match outcomes. For practical usage, the models were implemented in a Streamlit web application (33).

Using transfer learning, our model further advances the recent work of Scholtes and Karakuş (21). It can notably undergo an initial pre-training phase on a more extensive and historical dataset and a secondary training phase on a smaller, recent dataset to refine its predictive capabilities. The application of transfer learning within a Bayesian framework yielded results that were comparable to those of a commercial xG model (developed by StatsBomb), despite the utilization of a reduced dataset (see Table 2 for a summary). Furthermore, the interpretability of our linear model is a notable advantage as each model parameter can be analyzed and compared with one another. For instance, the *distance* from the goal had the most significant influence on xG predictions (over the shot *angle*) and should, consequently, be considered in any training drills and in-game tactical decision-making.

The Bayesian framework has several advantages. Building an efficient predictive model in a frequentist way implies training a model over a large enough dataset (34). In their study, Robberechts and Davis (35) concluded that five seasons of data were needed to fit an accurate frequentist and non-parametric xG model. Since the Bayesian inference comes with prior distributions, fewer training observations are needed to fit a model correctly. In addition, our approach also addressed confidentiality issues. Identifying a prior distribution implies learning a first function that approximates xG using a separated data sample (in our case, to use another competition to build the *baseline* model). Through transfer learning, models trained on multiple competitions can be reused for the targeted competition, reducing the computation time and improving model performances. Furthermore, retraining the baseline model on the competition of interest helps identify differences and similarities between the competitions.

In this work, we focused on open-play phases only, while overall, football performance should be modeled from all playing phases. However, the method is transferable to any playing phases where models could be built separately or combined. As aforementioned, player characteristics have significant importance in any football performance modeling. Player-specific data might improve model accuracy and outperform other commercial xG models. Gender has also shown some importance, as mentioned by Bransen and Davis (12). Considering gender in xG modeling or building gender or youth-specific models is recommended. Since football data on the women’s game are generally less abundant, the proposed approach based on transfer learning should address this issue as an optimal model would be built for a given competition while benefiting from broader information.

Even though sports performance modeling remains challenging due to its inherent complexity, analyzing players’ and teams’ characteristics provides essential information for this task. In this study, we considered individuals as teams since the data did not allow us to consider each player individually. Hence, the cross-random effects from the mixed logistic regression highlighted singularities between football teams, particularly regarding the *body_part_head* and *nb_opponent_traj* features. However, one may note that player-level information could significantly increase the performance of xG predictions, as players have strengths and weaknesses of their own. Beyond this, the model could allow for adapting a pre-game team strategy or making on-field decisions, to optimize the efficiency of the team.

Expert knowledge can be included in the model as priors. Nevertheless, if the number of observations is insufficient to construct the prior or if the prior information reflects extreme parameter values, there is a risk of bias and identification failure of posterior distributions (36–38). This phenomenon can be observed in our dataset when a comparison is made between the results of Scholtes’ model and the Bayesian xG model that we propose. Indeed, fitting the model by competition results in a smaller dataset than that employed in the aforementioned work. Furthermore, the selection of priors has led to a model with a higher xG prediction than that of StatsBomb and our own model. Conversely, when a sufficient number of observations have been accumulated, the incorporation of expert knowledge as a prior enables coaches to exert direct influence on predictions and decisions, thereby facilitating the provision of valuable real-time feedback. To illustrate, a coach’s knowledge of the game could be incorporated into the prior to facilitate meaningful insight into the players. Knowledge of the strengths of the starting team he has chosen, or of the opposing team, could lead to a manual shift in the value of the parameters influenced. Coaches could then readily utilize the model’s parameters to optimize in-game strategies, such as identifying the optimal shooting distance for individual players and offering practical insights for enhancing overall team performance.

The proposed method may also be of particular interest for modeling expected goals on target (xGOT). xGOT is a post-shot metric that uses the position at which the ball enters the goal and whether it is saved or scored. The xGOT is primarily employed to assess a team’s finishing proficiency by comparing xG with xGOT and to evaluate the performance of goalkeepers according to the quality of the shot. The methodology delineated in this paper also permits the identification of scenarios wherein specific teams exhibit superior finishing proficiency or more efficacious goalkeeping and the characterization of these scenarios. However, although our approach is generalizable to many cases, it is possible that predictive quality may be reduced in tasks that are too complex, such as expected threat. Maintaining high interpretability and, therefore, simple models for such tasks would potentially lead to a greater loss of predictive quality.

## 5 Conclusion

The development of interpretable and, more widely, explainable artificial intelligence represents a pivotal area of research within the field of computer science and subsequently sports science. This approach facilitates the extraction of novel insights from complex sports data, thereby empowering practitioners to make well-informed decisions (39). Our approach, based on a Bayesian mixed logistic regression model, is aligned with the principles of reproducibility and interpretability. Furthermore, it achieves comparable predictive performance to that of more complex models, despite the utilization of a limited sample of competition data. It also addresses practical concerns such as the identification of team strengths and weaknesses, and could be further extended to model xG from individual characteristics in a straightforward, accessible, and reliable manner.

## Data Availability

The original contributions presented in the study are included in the article/Supplementary Material, further inquiries can be directed to the corresponding author.
